# Variable phenotype expression in a family segregating microdeletions of the *NRXN1* and *MBD5* autism spectrum disorder susceptibility genes

**DOI:** 10.1038/s41525-017-0020-9

**Published:** 2017-05-03

**Authors:** Marc Woodbury-Smith, Rob Nicolson, Mehdi Zarrei, Ryan K. C. Yuen, Susan Walker, Jennifer Howe, Mohammed Uddin, Ny Hoang, Janet A. Buchanan, Christina Chrysler, Ann Thompson, Peter Szatmari, Stephen W. Scherer

**Affiliations:** 1 Department of Psychiatry and Behavioural Neurosciences, McMaster University, Hamilton, ON Canada; 2 Program in Genetics and Genome Biology, The Centre for Applied Genomics, The Hospital for Sick Children, Toronto, ON Canada; 3 Department of Psychiatry, University of Western Ontario, London, ON Canada; 4 Mohammed Bin Rashid University of Medicine and Health Sciences, Dubai, UAE; 5 Autism Research Unit, The Hospital for Sick Children, Toronto, ON Canada; 6 Centre for Addiction and Mental Health, The Hospital for Sick Children & University of Toronto, Toronto, ON Canada; 7 McLaughlin Centre and Department of Molecular Genetics, University of Toronto, Toronto, ON Canada

## Abstract

Autism spectrum disorder is a developmental condition of early childhood onset, which impacts socio-communicative functioning and is principally genetic in etiology. Currently, more than 50 genomic loci are deemed to be associated with susceptibility to autism spectrum disorder, showing de novo and inherited unbalanced copy number variants and smaller insertions and deletions (indels), more complex structural variants, as well as single-nucleotide variants deemed of pathological significance. However, the phenotypes associated with many of these genes are variable, and penetrance is largely unelaborated in clinical descriptions. This case report describes a family harboring two copy number variant microdeletions, which affect regions of *NRXN1* and *MBD5*—each well-established in association with risk of autism spectrum disorder and other neurodevelopmental disorders. Although each copy number variant would likely be categorized as pathologically significant, both genomic alterations are transmitted in this family from an unaffected father to the proband, and shared by an unaffected sibling. This family case illustrates the importance of recognizing that phenotype can vary among exon overlapping variants of the same gene, and the need to evaluate penetrance of such variants in order to properly inform on risks.

## Introduction

Autism spectrum disorder (ASD) is a developmental disorder of early childhood onset that impacts socio-communicative functioning, and which is principally genetic in etiology.^1^ It has a high rate of comorbidity with intellectual disability (ID) and other neurodevelopmental, neuropsychiatric, and medical disorders.^1^ ASD is relatively common, affecting ~1.5% of children,^2^ and is often associated with lifelong disability. Its core impairments and co-morbidities present a major challenge for caregivers and significant demands on health-care provision, and, by implication, health-care budget.^3^ Progress in elucidating its genetic etiopathogenesis will likely pave the way for new treatment options. To this end, significant progress has been made in the last decade with the advent of dense, high-throughput genotyping. More than 50 genes and loci harbouring de novo and inherited copy number variants (CNVs), structural variations, and single-nucleotide variants with diagnostic value (hereafter collectively referred to as ‘‘mutations’’ affecting the individuals discussed) have been implicated in ASD so far.^4, 5^ Functionally, many of these genes cluster in the post-synaptic density, whereas others are involved in neurite growth or histone modification.^6^

The term penetrance is used to describe the probability of a particular specified phenotype or set of phenotypes in those individuals harboring a particular mutation, whereas variable expression describes the range of phenotypic features observed among those with penetrant mutations.^7^ While some mutations are strongly associated with neurodevelopmental phenotypes such as intellectual disability and/or ASD,^8^ many of the mutations in the identified genes are characterized by incomplete penetrance, and variable expressivity and pleiotropy are seen in association with particular genotypes.^9^ For example, CNVs at 16p11–13 have been described in association with ASD in addition to a number of different neuropsychiatric disorders of variable severity,^10^ and the same is true of deletions in the *SHANK* ^11^ and *NRXN* genes.^12–15^ We are unaware of any susceptibility gene/locus that shows specificity for a single neurodevelopmental disorder.

Such variable expression and pleiotropy (multiple effects of a single gene) are not unusual, perhaps reflecting expression in different tissues, or shared pathophysiological mechanisms between disorders.^7, 16^ The ultimate phenotype may be influenced by the interplay of these with other factors:genetic (including sex), environmental (including maternal and hormonal influences) and epigenetic.^7^ A more striking observation is that some variants, classified as pathogenic, can be present without any apparent clinical consequence in some people—i.e., non-penetrant. In particular, some individuals with ASD or other neurodevelopmental disorders have been reported to share an ostensibly pathogenic variant with a phenotypically normal transmitting parent, and sometimes also one or more unaffected siblings.^17–19^ The underlying mechanism illustrated by such cases is poorly understood, although factors such as genomic context, impact on protein structure and function, and the effect of modifier genes may be important.^7^

Family based research does indicate that ASD itself is often expressed as a broader phenotype, beyond the bounds of the clinical spectrum, with family members often displaying mild, subclinical, traits.^20^ Indeed, the sibling recurrence for this broad autism phenotype is higher than for ASD.^21^ These milder traits do not often impact function, and, therefore, may not be immediately apparent or come to the attention of clinical services, but their importance lies in their implication for our understanding of the biology of ASD, and the penetrance and expression of the underlying genes.

We performed extensive genetic analyses including whole-genome sequencing in an individual with ASD and his family. We identified CNV deletions involving *NRXN1* and *MBD5* in the proband, but also in his father and sister, neither of whom had evidence of any clinically overt brain-related phenotype. *NRXN1* and *MBD5* are implicated in ASD, ID, and other neuropsychiatric disorders,^12, 13, 22, 23^ and functional mutations of either gene might be expected to have phenotypic consequences. We noted other variants of potential relevance to the ASD phenotype. This family illustrates that mutations anticipated to be highly penetrant may in fact be less so, and at times, apparently without phenotypic consequence.

## Results

### Clinical characteristics of family

**Fig. 1:**
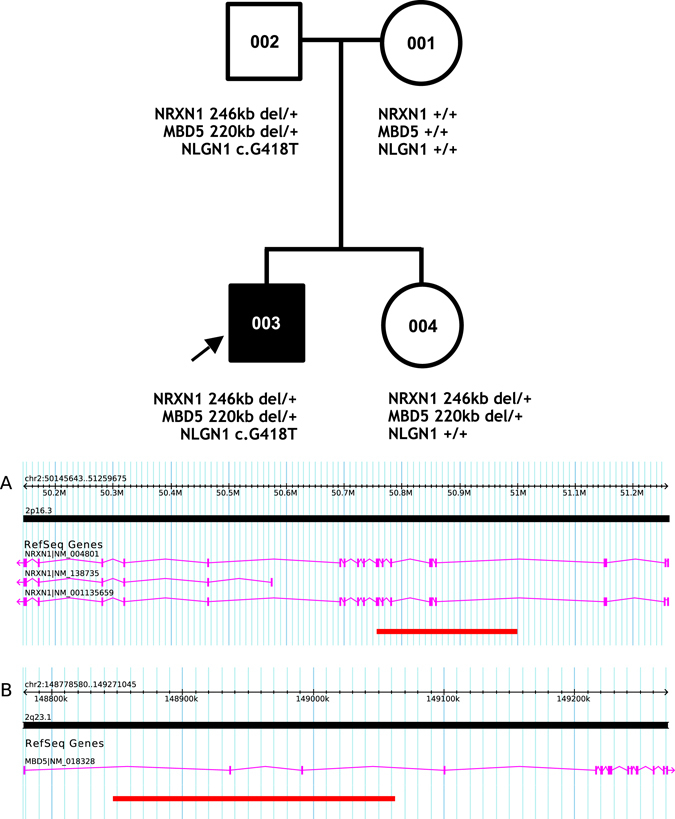
Pedigree with microarray results and annotated *NRXN1* (**a**) and *MBD5* (**b**) CNVs

The proband (003) (Fig. 1) was diagnosed with attention deficit hyperactivity disorder (ADHD) at age 3 years and with ASD at age 5 years through a specialty ASD clinic. His mother and father were aged 33 and 34 years, respectively, at the time of his conception, and the mother reported a medically uneventful pregnancy. She had no history of miscarriage, and the male proband, her firstborn, was born by vaginal delivery following spontaneous labor at 39 weeks gestation. Birth weight was 4054 g. There were no neonatal complications, and no craniofacial dysmorphology noted. Development during the 1st year was normal, but by 36 months he began losing acquired language, and speech became echolalic and scripted. Although gross motor control was intact, fine motor was an additional area of early developmental difficulty. By 36 months, repetitive motor mannerisms and preoccupations become prominent. Assessment of intellectual ability and adaptive functioning were consistent with a diagnosis of intellectual disability, and in the specialty clinic an additional diagnosis of ADHD was made (Table 1). Further assessment in the clinic at the age of 5 years identified more significant socio-communicative vulnerabilities, and a diagnosis of ASD was given. At that time an Intelligence Quotient (IQ) of 53 was recorded (verbal IQ = 56, non-verbal IQ = 65), and his adaptive skills were largely consistent with function in the mildly impaired range (adaptive composite = 63). At age 12 years 10 months his head circumference was 57.9 cm, consistent with macrocephaly.

**Table 1:** Summary of family’s genotypes and phenotypes

	−001	−002	−003	−004
Sex	Female	Male	Male	Female
Microarray	NRXN1 +/+ MBD5 +/+	NRXN1 del/+ MBD5 del/+	NRXN1 del[pat]/+ MBD5 del[pat]/+	NRXN1 del[pat]/+ MBD5 del[pat]/+
WGS	–	NLGN1, c.G418T [p.D140Y]	ASB14, c.461 462del [p.L154fs] NLGN1, c.G418T [p.D140Y]	Targeted Sanger: NLGN1 +/+
Clinical diagnosis	None	None	ASD, ID, ADHD	None
IQ	NA	NA	WPPSI-IIIF	WASI
	–	–	FSIQ = 53,	FSIQ = 121,
	–	–	VIQ = 56,	VIQ = 124,
	–	–	NVIQ = 65	NVIQ = 112
Vineland adaptive behavior scales (comm = communication, daily = daily living)	NA	NA	Comm 3%ile, daily 1%ile, social <1%ile	NA
Morphology (*HC* = head circumference)	HC = 56 cm, height = 166.4 cm, weight = 60.3 kg (age 45 years)	HC = 58 cm, height = 182.9 cm, weight = 83.9 kg (age 44 years)	HC = 57.9 cm, height = 166.5 cm, weight = 58.1 kg (age 12:10)	HC = 51 cm, height = 106.7 cm, weight = 17.7 kg (age 6:6)
Medical	Nil	Nil	Nil	Nil
Language	CC-A:	CC-A:	OWLS:	CCC:
	Language 42%ile, pragmatics 7%ile, social engagement 7%ile	Language 7%ile, pragmatics 9%ile, social engagement 19%ile	Standard score = 50 (<0.1%ile) CCC: GCC = 30 (1%ile)	GCC = 71 (32%ile)
SRS	*T*-score = 61 (normal)	*T*-score = 43 (normal)	NA	*T* score = 45 (normal)
PPVT	109 (73%ile)	101 (%ile)	86 (9%ile, aged 7:4)	73 (86%ile, aged 3:7)
RMET	31 (86% correct)	19 (53% correct)	NA	NA

*NA* not available, *CC-A* communication checklist–adult, *CCC* children's communication checklist, *OWLS* oral and written language scales, *SRS* social responsiveness scale, *PPVT* Peabody Picture Vocabulary Test, *RMET* Reading the Mind in the Eyes Test, *FSIQ* fullscale IQ, *VIQ* verbal IQ, *NVIQ* nonverbal IQ

The proband’s younger sister (004) was born following an uneventful pregnancy by normal vaginal delivery. Birth weight was 3856 g. She required incubation and monitoring for transient tachypnea, which resolved spontaneously, but otherwise there were no perinatal complications. Her early language and motor milestones were attained without delay. She did experience stuttering at 36 months, but otherwise exhibited no social or communicative vulnerabilities. Her cognitive function was in the superior range on the Wechsler Abbreviated Scale of Intelligence (WASI-II) age 6 years, and she had above average expressive and receptive language skills. Her scores on the Autism Diagnostic Interview-Revised (ADI-R) and the Autism Diagnostic Observation Schedule (ADOS, module 3), both completed when she was aged 9 years, did not indicate ASD symptoms. Similarly, neither children’s communication checklist (CCC)^24^ nor the child version of the social responsiveness scale (SRS)^25^ revealed any such developmental vulnerabilities.

We evaluated both parents (001 and 002) for the presence of neurocognitive vulnerabilities and neuropsychiatric diagnoses (Table 1). Both graduated high school and attained professional level employment. The SRS was not consistent with any ASD traits in either parent, although the communication checklist—adult (CC-A)^26^ indicated some maternal and paternal communication vulnerabilities, and father’s score on the Reading the Mind in the Eyes Test (RMET)^27^ was below average, suggesting some impairment in theory of mind abilities. No additional social or communication vulnerabilities were apparent, and neither parent had findings consistent with ASD. Moreover, besides maternal post-natal depression, both parents denied any neuropsychiatric history.

### Genetic characterization of family

All four family members provided blood for genotyping. We initially identified hemizygous microdeletions in the chromosomal regions 2p16.3 (50,754,487–50,996,179 [hg19]) and 2q23.1 (148,851,175–149,059,335 [hg19])] in both offspring and their father. By microarray, we estimated the deletion at 2p16.3 to be ~242 kb eliminating exons 6 to 16 of *NRXN1* (Fig. 1). The 2q23.1 deletion was ~215 kb in size, eliminating non-coding exons 2 and 3 in the 5’ untranslated region (UTR) of *MBD5* (Fig. 1). We validated both deletions using SYBR-Green based real-time quantitative PCR. We found no other CNVs deemed clinically significant or of uncertain clinical significance according to the American College of Medical Genetics’ guidelines,^28^ in any family member.

We undertook whole-genome sequencing of the proband and both parents using the BGI platform as previously described.^29^ This validated the *NRXN1* and *MBD5* deletions. The breakpoints of the *NRXN1* deletion were further mapped to 50,754,222–51,000,379 [hg19] by Sanger sequencing, and visual inspection of BAM files mapped the *MBD5* breakpoints to 148,843,025- 149,062,962 [hg19], thereby adjusting the size of the *NRXN1* and *MBD5* deletions to ~246 and ~220 kb, respectively. There were no additional structural alterations at the breakpoints. In the proband and his father we found a missense variant (c.G418T [p.D140Y]) in *NLGN1* (an ASD risk gene), which was predicted by in silico algorithms to be damaging. Targeted Sanger sequencing of the unaffected sibling’s DNA did not identify the variant. Finally, the proband had a de novo 2 bp deletion (c.461_462del [p.L154fs]) involving *ASB14*. Although the mutation was predicted to lead to a frameshift of the protein, this gene has not been associated with ASD or other neurodevelopmental disorders, to date. We identified no other rare loss-of-function or de novo missense SNVs this family’s genomic sequences.

### Prevalence and penetrance of overlapping mutations

We next examined clinical and population data sets to investigate the penetrance of the putative mutations identified. For *NRXN1* and *MBD5*, we specifically focused on CNV deletions with at least a 50% reciprocal overlap with that of the proband (hereafter termed ‘‘overlapping CNVs’’). First, we examined clinical data sets comprising individuals (*N* = 19,237, comprising *N* = 5273 cases and their family members) ascertained by way of one or more different neurodevelopmental diagnoses, including ASD,^6, 30^ developmental delay,^31^ OCD^32^ and cerebral palsy (CP).^19^ These individuals had been genotyped on a variety of platforms, each allowing a CNV detection threshold of 10 kb using five or more probes. Only CNVs called with two or more algorithms were considered. Thus, we identified one clinical case with an overlapping *NRXN1* CNV (chr2: 50,761,808–51,037,134 [hg19]), and two clinical cases with overlapping *MBD5* CNVs (chr2: 148,787,060- 149,106,568 and 148,842,503–149,059,335 [hg19]) from a total of 6 and 7 exon-impacting *NRXN1* and *MBD5* CNVs, respectively. All three individuals have developmental delay but no further phenotype information was available. We next examined population data sets, comprising samples genotyped on the Illumina 2.5M platform (KORA and COGEND)^33, 34^ and Illumina 1M platform (WTCCC, SAGE, ONC, and HABC),^35–37^ giving rise to a total sample size of 13,871. A CNV detection threshold of 30 kb was employed using a minimum of five probes. We considered all CNVs called by two or more algorithms, identifying from this pooled data set one *NRXN1* CNV and one *MBD5* CNV from a total of 61 (six exonic) and 19 (seven exonic) *NRXN1* and *MBD5* CNV deletions, respectively. Consequently, there was no statistical evidence for a greater prevalence of overlapping *NRXN1* or *MBD5* CNVs among cases than among controls.

**Fig. 2:**
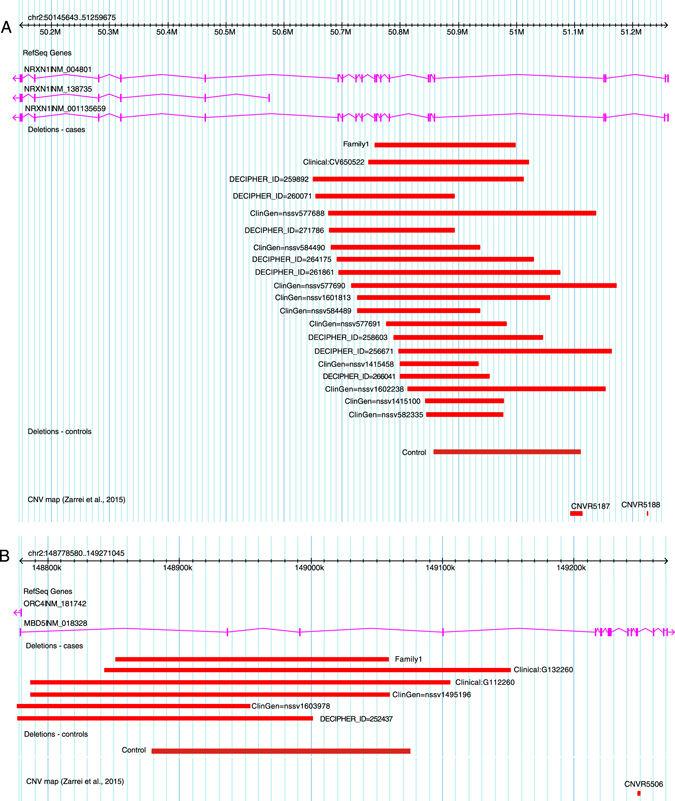
CNVs overlapping the family’s *NRXN1* (**a**) and *MBD5* (**b**) CNV deletion from clinical (‘‘clinical’’) and population (‘‘controls’’) data sets, and DECIPHER and ClinGen

We also considered overlapping CNVs recorded in DECIPHER—a clinician-submitted sample of 21,688 individuals with identified phenotypes and validated CNVs.^8^ Of this sample, eight individuals had overlapping *NRXN1* CNVs, and one an overlapping *MBD5* CNV (Fig. 2). Similarly, the International Standards for Cytogenetic Arrays (ISCA) clinical database^38^ included 11 overlapping *NRXN1* CNVs and 8 overlapping *MBD5* CNVs. The phenotypes described among these 19 individuals all included ID with an additional diagnosis of ASD in five. Among these CNVs, DECIPHER inheritance pattern is described as de novo for the one *MBD5* deletion, and variable for the *NRXN1* deletions (de novo for 3, inherited for 3, and unknown for 2).

We also examined our whole-genome sequenced ASD families (comprising *N* = 5205 probands, siblings and parents, see methods) for any additional individuals with damaging *NLGN1* variants. We found overlapping variants in three individuals, two of with ASD, and one an unaffected father. Exome Aggregation Consortium (ExAC),^39^ a data set which spans 60,706 unrelated individuals sequenced as part of various disease-specific and population genetic studies, lists two individuals with an overlapping mutation.

## Discussion

In the family described here, CNV deletions in two ASD-implicated genes, *NRXN1* and *MBD5*, are shared by an ASD proband, his typically developing sibling, and their unaffected father. Based on extensive literature,^40^ our clinical diagnostic laboratory would have assigned either one of these CNVs as ‘‘likely pathogenic’’ for ASD. In addition, WGS identified a missense variant in the putative ASD gene *NLGN1* that was paternally transmitted to only the proband. This is of interest for the known interaction between the NLGN1 and NRXN1 proteins,^41^ and in this family the missense mutation was predicted to be damaging. A de novo frameshift variant in *ASB14* was also identified in the proband, and although not brain expressed, we cannot rule out an etiological role for this mutation.

NRXN1 is one of three neurexin scaffolding proteins; aberrations in its gene are strongly associated with cognitive, neurodevelopmental and neuropsychiatric phenotypes.^12, 13^ Deletions in *NRXN1* are relatively common in ASD (0.45%) and ID (0.12%) cohorts,^12^ but much less frequently seen in population based surveys (~0.02%) (ref. 12). Other studies have also highlighted the fact that deletions can occur in apparently healthy individuals (ref. 12 and references therein). An estimate of penetrance for all CNVs for schizophrenia and neurodevelopmental phenotypes for NRXN1 is 33% (ref. 42). The ExAC constraint metric for this gene is −0.13 (ref. 39), which is consistent with tolerance to copy number variation. However, its low burden of mutations in the healthy population coupled with high expression levels (messenger RNA and protein) results in its categorization as critical to brain development.^43^ *MBD5* encodes a methylated-DNA binding protein, which has previously been described in the literature as highly penetrant, characterized in all cases by intellectual disability, ASD and, more variably, craniofacial abnormalities.^22, 23^ Most of the cases described so far have been de novo, although transmission is sometimes unknown. In the present family, the proband’s phenotype is largely consistent with previous descriptions connected with mutations of NRXN1 and MBD5,^12, 13, 22, 23^ characterized by moderate ID and ASD in the absence of dysmorphism. The ExAC constraint measure for *MBD5* is 0.69 (ref. 39) indicating some degree of intolerance to variation, although it is not classified critical to brain development.^43^

Beyond the gene *per se*, the exact genomic location of a CNV may be important in the determination of functional aberration and phenotypic consequence. In this family, the 2p16.3 deletion impacted exons 6–16, and our analysis indicated that overlapping CNVs were rare among clinical cases or population controls. Crucially, overlapping CNVs were not enriched among the cases compared with controls. We speculate that this hemizygous deletion impacting only exons 6–16 may be less penetrant than others reported for this gene. Indeed, most clinical cases seem to cluster around exons 1–4 at the 5’ end of the gene, with deletions that impact the subsequent exons showing evidence of lower penetrance.^13^ This may be due to influence of the lncRNA AK127244 adjacent to the promotor of alpha-*NRXN1*.^13^ Confounding the argument of lower penetrance, however, is the 20 individuals with overlapping CNVs in ISCA, DECIPHER, and the other clinical data sets we examined with variable but largely overlapping phenotypes.

Similarly, overlapping *MBD5* deletions were not enriched among clinical cases, although mutations described largely overlap with that of our patient, impacting one or more exons in the 5’-UTR. Two additional individuals in our clinical data set, with developmental delay, had identical *MBD5* CNVs. Although these exons are not translated, all 5’-UTR deletions result in haploinsufficiency, with peripheral expression of *MBD5* approximately halved.^23^ Many cases described, including the one in DECIPHER, have de novo mutations.

Finally, *NLGN1*, is of potential interest, forming complexes with *NRXN1*, and implicated in both structural integrity and function of synapses.^44^ While the function of *NLGN1* has been well described, particularly in the context of its interaction with *NRXN1*, the phenotype associated with gene mutations has not been elaborated. One genome-wide CNV analysis of ASD cases identified enrichment for CNVs in *NLGN1* compared with population controls,^45^ and another provided evidence of association between common variants in *NLGN1* and schizophrenia in the Han Chinese population.^46^ However, the penetrance of the mutation described in our family is unclear in light of the identification of a similar number of cases in our clinical data set and ExAC.

Complexity of the etiology underlying ASD is well demonstrated in families like that presented here, where the most advanced genomic technologies have provided a comprehensive genetic profile, and the variants detected are shared among family members with and without ASD. We are reminded to acknowledge what remains unknown (e.g., the role of environmental factors and epigenetic regulation) and not to overstate the causal impact of variants. We are spurred to investigate the mechanisms whereby genotype can lead to phenotype in some, but not others.^47^ Variously, this may be a function of the type of variant (i.e., loss-of-function, missense, deletion), its location (i.e., exonic, intronic, regulatory region, intergenic), or the resultant transcript/isoform.^9^ We must, however, move from a genetic to a genomic perspective, recognizing that no gene or gene product functions in isolation. Indeed, each of the three genetic aberrations in this family might have been deemed sufficient to explain ASD in the proband, but all were non-penetrant in other family members. For future investigations related to penetrance, we recommend the approach of comparing only highly overlapping CNVs, rather than all CNVs involving the same gene. A true estimate of penetrance will require a more robust approach than ours, with access to comprehensive control data from pedigrees^48^ and large data sets.^49^ For example, although many variants may be very rare in the population, those that are inherited can be tracked through family members and their segregation with disease phenotype examined. This allows a quantification of their pathogenicity to be determined,^48^ as well as a Bayesian Factor to be estimated, which can be used as a test of the hypothesis of causality by examining its distribution under the hypothesis of neutrality.^50^ Although not a direct measurement of penetrance per se, this approach does go some way to quantifying the probability of disease in association with particular mutations.

Context is crucial. The impact of the wider genomic landscape, including the epigenome, along with factors such as age, sex and the early environmental milieu, will undoubtedly contribute to a person’s evolving phenotypes. The rich tapestry of protein interactions at the cellular level translate more proximally into endophenotypes, which, rather than global diagnostic fields, are the more internal phenotypic elements or markers revealed by specific measures. In the family presented here, the father’s vulnerabilities decoding emotions from facial stimuli may, for example, represent such an endophenotype. The ‘‘vulnerable brain’’ may be impacted by another factor to result in the full expression of a clinical phenotype. These mechanisms will become untangled as a result of large, epidemiological studies, but also the accumulation of evidence from case studies such as this one.

## Methods

The family described was recruited as part of ongoing studies of the genetics of ASD (www.mss.ng).^51^ This data set currently comprises ~2500 probands with ASD and, in most cases, both parents. ASD diagnoses are made by expert clinicians using the ADI-R and the ADOS combined with clinical judgment. Probands and their available first degree relatives have all undergone phenotyping as described below, and have provided DNA for the identification of CNVs and SNVs (see below). All data were collected following informed consent from participants or substitute decision makers, and the study is conducted with approval from respective local research ethics boards. The family described in detail in this paper has provided specific written consent for their data to be shared in the scientific literature in the form of this case report.

### Phenotypes

In addition to the ADI-R and ADOS-G, the proband underwent a cognitive assessment using the Wechsler Preschool and Primary Scale of Intelligence (WPPSI) and a language assessment with the oral and written language scales (OWLS-II). Additionally, measures of the proband’s adaptive functioning (Vineland Adaptive Behavior Scales-II) was completed with his parents. Both parents were assessed using the Peabody Picture Vocabulary Test and the RMET (a measure of theory of mind).^27^ In addition, both parents completed the CC-A^26^ and the SRS.^25^ The proband’s sibling underwent assessment with the WASI-II, the ADI and the ADOS. Her parents completed a measure of her social communication (CCC).^24^ Height, weight, and head circumference were measured for each family member.

### Genotypes

We called CNVs as previously described.^31^ Briefly, four different CNV calling algorithms were used to annotate high-confidence CNVs. These included the Affymetrix Chromosome Analysis Suite (ChAS), iPattern,^52^ Nexus,^53^ and Partek.^54^ A stringent set of variants was defined for further analyses. This set included variants detected by one or both of ChAS or iPattern, and if detected by only one of these, then also by one of Nexus or Partek. For stringent calls on the X chromosome, we required calling by both ChAS and iPattern. Only CNVs with five probes or more on the array were called, with a minimum length cutoff of 30 kb. CNVs were filtered to prioritize rare variants that occurred with a frequency of<0.1% in control samples (*N* = 9611). For the purpose of filtering, CNVs with >50% reciprocal overlap were deemed overlapping. We also removed all CNVs that had >70% overlap with a known segmental duplication. We further restricted our list to those with more than 75% overlap with copy number stable regions, according the stringent CNV map of the human genome.^55^ All CNVs described in the index family have been validated using the SYBR green based quantitative PCR method. The genomic coordinates presented in this paper are based on the February 2009 Human Genome Build (GRCh37/hg19).

The proband and both parents also underwent whole-genome sequencing (WGS) by BGI as described previously.^29^ All identified variants were subsequently validated by Sanger sequencing. In addition, the unaffected sibling underwent targeted Sanger sequencing. We annotated the Identified SNVs, and prioritized those likely to be damaging using a filtering algorithm. This captured all those SNVs that were rare (≤1% minor allele frequency), and involved loss of function (nonsense, splice site, and frameshift), and damaging de novo missense mutations (damaging as evidenced by two of the following criteria: SIFT ≤ 0.05, Polyphen2 ≥ 0.95, CADD ≥ 15, Mutation Assessor score ≥ 2, placental mammal PhyloP ≥ 2.4 and vertebrate PhyloP ≥ 4).^29^

### Data availability

Sequence data has been deposited at the European Genome–phenome Archive (EGA, http://www.ebi.ac.uk/ega/), which is hosted by the EBI, under accession number EGAS00001001023. The data, as part of a larger autism whole-genome sequencing project, are also available in the MSSNG database on Google Genomics (for access see http://www.mss.ng/researchers).
